# Infection of *Porphyromonas gingivalis* Increases Phosphate-Induced Calcification of Vascular Smooth Muscle Cells

**DOI:** 10.3390/cells9122694

**Published:** 2020-12-15

**Authors:** Hyun-Joo Park, Yeon Kim, Mi-Kyoung Kim, Hae-Ryoun Park, Hyung-Joon Kim, Soo-Kyung Bae, Moon-Kyoung Bae

**Affiliations:** 1 Department of Oral Physiology, BK21 PLUS Project, School of Dentistry, Pusan National University, Yangsan 50610, Korea; phj3421@hanmail.net (H.-J.P.); graceyeon88@gmail.com (Y.K.); eenga@naver.com (M.-K.K.); hjoonkim@pusan.ac.kr (H.-J.K.); 2 Periodontal Disease Signaling Network Research Center (MRC), School of Dentistry, Dental Research Institute, Pusan National University, Yangsan 50610, Korea; parkhr@pusan.ac.kr (H.-R.P.); skbae@pusan.ac.kr (S.-K.B.); 3 Dental and Life Science Institute, School of Dentistry, Pusan National University, Yangsan 50610, Korea; 4 Department of Oral Pathology, School of Dentistry, Pusan National University, Yangsan 50610, Korea; 5 Department of Dental Pharmacology, BK21 PLUS Project, School of Dentistry, Pusan National University, Yangsan 50610, Korea

**Keywords:** *Porphyromonas gingivalis*, vascular smooth muscle cells, vascular calcification

## Abstract

Accumulating evidence suggests a link between periodontal disease and cardiovascular diseases. Vascular calcification is the pathological precipitation of phosphate and calcium in the vasculature and is closely associated with increased cardiovascular risk and mortality. In this study, we have demonstrated that the infection with *Porphyromonas gingivalis* (*P. gingivalis*), one of the major periodontal pathogens, increases inorganic phosphate-induced vascular calcification through the phenotype transition, apoptosis, and matrix vesicle release of vascular smooth muscle cells. Moreover, *P. gingivalis* infection accelerated the phosphate-induced calcium deposition in cultured rat aorta ex vivo. Taken together, our findings indicate that *P. gingivalis* contributes to the periodontal infection-related vascular diseases associated with vascular calcification.

## 1. Introduction

Periodontal pathogens present in the subgingival plaque biofilms can cause complex chronic inflammatory disorders of the periodontium [1]. *Porphyromonas gingivalis*, a gram-negative black pigmented anaerobic bacterium, is a well-known and important pathogen linked to the initiation and progression of periodontal diseases [2]. Accumulating evidence indicates that periodontal diseases are a potential risk factor for several systemic diseases, including diabetes, rheumatoid arthritis, and cardiovascular diseases, in addition to preterm birth and low birth weight [3]. Among these, the incidence of cardiovascular diseases, in particular, is strongly correlated with *P. gingivalis* infection [4]. Several in vitro experiments and studies using animal models have demonstrated that *P. gingivalis* and its virulence factors are involved in the pathogenesis at various stages of atherosclerosis, like endothelial dysfunction, intimal hyperplasia, foam cell formation, and vascular remodeling [5].

Vascular calcification, characterized by pathological deposition of calcium phosphate in vasculatures, is a risk factor for cardiovascular events and is commonly associated with aging, diabetes mellitus, atherosclerosis, and chronic renal disease [6]. It is a complex and actively regulated process, which results in the phenotypic transformation of vascular smooth muscle cells (VSMCs) into osteoblast-like cells [7]. Experimental and clinical studies have shown that elevated levels of phosphate contribute to the pathogenesis of vascular calcification in chronic renal disease, through osteoblastic differentiation of VSMCs [8]. Virulence factors such as lipopolysaccharide (LPS) and outer membrane vesicles derived from *P. gingivalis* stimulate the proliferation and calcification of VSMCs [9,10]. Periodontal pathogens, including *P. gingivalis*, from the inflamed periodontal tissues can disseminate into the systemic circulation, [11] circulate freely or within cell autophagosomes, reach distant tissues or organs, including arteries, and invade vascular cells, including arterial endothelial cells and arterial SMCs [12,13]. The direct effect of periodontogens such as *P. gingivalis* on vascular calcification induced by phosphate has not been clearly defined yet. In the present study, we studied the detrimental effects of *P. gingivalis* on the phosphate-induced calcification of VSMCs and on the organ culture model of rat aorta to understand the underlying mechanisms.

## 2. Materials and Methods

All animal studies were conducted in accordance with the Guide for the Care and Use of Laboratory Animals (NIH publication No. 85-23 revised 1996) and approved by the Institutional Animal Care and Use Committee at Pusan National University, Korea (PNU-2018-2037).

### 2.1. Reagents and Antibodies

Antibodies for Runx2, phospho-Smad1/5, phospho-Akt, Akt, total/cleaved caspase-3, Bcl2, and Bad were purchased from Cell Signaling (Danvers, MA, USA). Rabbit polyclonal anti-Calponin and anti-Gas6 antibodies were purchased from Abcam (Cambridge, UK). Rat-specific anti-β-actin and anti-α-tubulin antibodies were purchased from Bioworld Technology (St. Louis Park, MN, USA). Smad1/5, Axl and HRP-conjugated goat anti-mouse and anti-rabbit IgG were procured from Thermo Fisher Scientific (Waltham, MA, USA). 

### 2.2. Bacterial Strain and Growth Conditions

*P. gingivalis* strain 381 (origin: FCD 381) was purchased from American Type Culture Collection (ATCC; BAA-1703, Manassas, VA, USA). *P. gingivalis* strain 381 was cultured in gifu anaerobic medium (GAM) broth (Nissui Pharmaceutical, Tokyo, Japan), which contained vitamin K (5 µg/mL) and hemin (5 µg/mL) at 37 °C in an anaerobic chamber in an atmosphere containing 90% N_2_, 5% H_2_, and 5% CO_2_. An optical density (OD) of 1.0 (660 nm) was found to correspond to 10^9^ colony forming units/mL. To prepare the bacteria for infection, an overnight culture was diluted to 1.0 OD at 660 nm in GAM, washed and resuspended in phosphate buffered saline (PBS), and used to infect the cells at a multiplicity of infection (MOI) of 100.

### 2.3. Cell Culture

To isolate VSMCs, male Sprague–Dawley rats (3 weeks old, 40~60 g, Samtaco, Osan-si, Gyeonggi-do, Korea) were euthanized using intraperitoneal injection of sodium pentobarbital (60 mg/kg). The thoracic aorta was cut out and the surrounding fat and connective tissues were discarded. It was slit longitudinally and its lumen surface was scraped with a razor blade to remove the intima, before cutting it into 3–5 mm long pieces. It was explanted lumen side down on collagen-coated culture dishes. After seven days, tissue fragments were discarded and sprouted VSMCs were collected (referred to as P0). A7r5 cells, purchased from the ATCC (CRL-1444^™^), and primary VSMCs were grown in Dulbecco’s modified Eagle’s medium (DMEM, Thermo Fisher Scientific, Waltham, MA, USA) with 10% fetal bovine serum (FBS, Thermo Fisher Scientific) and 1% antibiotics (Thermo Fisher Scientific), at 37 °C in 95% humidified air with 5% CO_2_. Primary VSMCs were used for flow cytometry analysis and A7r5 cells were used for other experiments. 

### 2.4. Infection of Cells with P. gingivalis 

A7r5 cells were cocultured with live *P. gingivalis* strain 381 at 37 °C (MOI 1:100). After 3 h, the cells were washed with PBS and cultured in fresh medium. For control, A7r5 cells were subjected to medium change and PBS wash but without bacteria. 

### 2.5. Carboxyfluorescein Succinimidyl Ester (CFSE) Staining

*P. gingivalis* strain 381 suspension was incubated with 5 μM CFSE (Molecular Probes, Eugene, OR, USA) in PBS for 15 min and washed twice in PBS. After 3 h of infection of VSMCs with CFSE-tagged *P. gingivalis* was confirmed by observation under a confocal microscope (LSM510; Carl Zeiss, Germany).

### 2.6. Induction and Quantification of Calcification

A solution of Pi (Na_2_HPO_4_ and NaH_2_PO_4_, pH 7.4) was added to growth medium to get effective concentrations of 1.4, 2.6, and 3.5 mM. The cellular ALP activity and calcium content were determined using a ALP assay kit (Takara, Los Angeles, CA, USA) and calcium colorimetric assay kit (BioVision, Milpitas, CA, USA), respectively. To extract proteins, cells were solubilized in 0.1 M NaOH with 0.1% SDS, and the protein content was measured by using a Bio-Rad protein assay kit.

### 2.7. ARS Staining

To observe calcium deposition, cells were fixed with 4% paraformaldehyde at room temperature for 20 min and stained with 2% alizarin red S solution (pH 4.2, adjusted with 1.0% NH_4_OH) for 10 min at room temperature. After staining, calcium deposits were photographed. After imaging, stained cells were destained with 10% acetic acid for 20 min, and the calcium concentration was measured based on the absorbance at 420 nm using an ELISA reader (Dynex, Lincoln, UK).

### 2.8. Von Kossa Staining and Quantification

Von Kossa staining and quantification were performed according to a previously described method [14]. Briefly, aortic tissues were treated with a 1% silver nitrate solution under a UV lamp for 15 min, soaked in 5% sodium thiosulfate for 5 min. The photomicrographs of stained calcium deposits were analyzed using a software developed in-house using MATLAB 2019 (Math Works).

### 2.9. Quantitative Real-Time RT-PCR

Real-time PCR quantification (qRT-PCR) was performed using SYBR^®^ Green (Light Cycler; Roche Applied Science, Penzberg, Germany). Cycling parameters included 1 cycle at 95 °C for 10 min, for denaturation, followed by amplification for 30 cycles at 95 °C for 10 s, 60 °C for 5 s, and 72 °C for 7 s. Subsequently, a melting curve program was applied with continuous fluorescence measurement and data analysis was monitored using Light Cycler software (version 4.0). The sequences of oligonucleotide primers used are shown in Supplementary Table S1.

### 2.10. Western Blot Analysis

Harvested cells were lysed in a buffer containing 40 mM Tris-Cl, 10 mM EDTA, 120 mM NaCl, 0.1% Nonidet P-40, and a protease inhibitor cocktail (Sigma, St. Louis, Mo, USA), and the protein concentration was measured by the BCA assay. Cell lysates (30 μg/lane) were separated on SDS–polyacrylamide gels and transferred onto a nitrocellulose membrane (GE Healthcare Life Sciences, Pittsburgh, PA, USA). The membrane was blocked with 5% non-fat milk for 60 min and probed with the appropriate antibodies. Protein bands were detected using an enhanced chemiluminescence detection system (GE Healthcare Life Sciences).

### 2.11. Immunocytochemistry

Cells were fixed with 4% paraformaldehyde for 10 min, blocked with 1% normal goat serum in PBS-T for 60 min, and incubated first with appropriate primary antibodies followed by incubation with Alexa^®^488- and 594-conjugated secondary antibodies (Life Technolgies, Carlsbad, CA, USA). Coverslips were mounted in Vectastain containing DAPI (Vector Laboratories, Burlingame, CA, USA), and the images were captured and analyzed by fluorescence microscopy (Nikon, Minato, Tokyo, Japan).

### 2.12. Flow Cytometry Analysis

*P. gingivalis* infected primary VSMCs were incubated in the calcification medium for five days, washed twice in 1X PBS, and fixed in chilled 70% ethanol. The cells were stained with 5 μg/mL propidium iodide (Sigma) at room temperature for 10 min and analyzed using a FACS Calibur flow cytometer (BD Bioscience, San Jose, CA, USA). The cell cycle profile was analyzed using the Modfit LT software.

### 2.13. TUNEL Assay

Apoptotic cells were detected using the Dead EndTM Fluorometric TUNEL System (Promega, Madison, WI, USA) in accordance with the manufacturer’s instructions. Cells were incubated in calcification medium for 5 days, fixed in 4% paraformaldehyde for 25 min at 4 °C, and permeabilized with 0.2% Triton X-100 for 5 min at room temperature. Free 3′ ends of fragmented DNA were enzymatically labelled with the TdT-mediated dUTP nick end labelling (TUNEL) reaction mixture for 60 min at 37 °C in a humidified chamber. Labelled DNA fragments were visualized under a fluorescence microscope (Nikon).

### 2.14. Matrix Vesicle Isolation

Matrix vesicles were harvested following a modified matrix vesicle isolation protocol [15]. *P. gingivalis* infected A7r5 cells were washed twice with PBS and transferred to control or calcification medium, digested with collagenase, and centrifuged at 10,000× *g*. Matrix vesicles were then harvested from the supernatant after centrifugation at 100,000× *g* for 60 min at 4 °C in an ultracentrifuge (Beckman, Brea, CA, USA). Matrix vesicles were resuspended in 1% Triton X-100, and protein concentration and ALP activity were determined. 

### 2.15. Arterial Ring Calcification

Pieces of the aorta from the thoracic to the iliac arteries of the male Sprague–Dawley rats (6 weeks old, 150–200 g, Samtaco) were resected in a sterile manner. After removing the adventitia and endothelium, the vessels were cut into 2–3 mm rings and placed in either calcification medium or normal culture medium at 37 °C in 5% CO_2_ for 10 days, with medium changes once every three days. 

### 2.16. Statistical Analysis

All statistical analyses were carried out using the Sigma Plot 11.0 Software (Systat Software Inc., Erkrath, Germany), and all values are expressed as mean ± S.D. Differences between selected pairs from the experimental groups were analyzed for statistical significance using the paired sample Student’s t-test and one-way ANOVA followed by Bonferroni testing. The statistical difference was considered to be significant if the *p*-value was < 0.05.

## 3. Results

### 3.1. P. gingivalis Promotes Inorganic Phosphate (Pi)-Induced Calcium Deposition in VSMCs 

**Figure 1:**
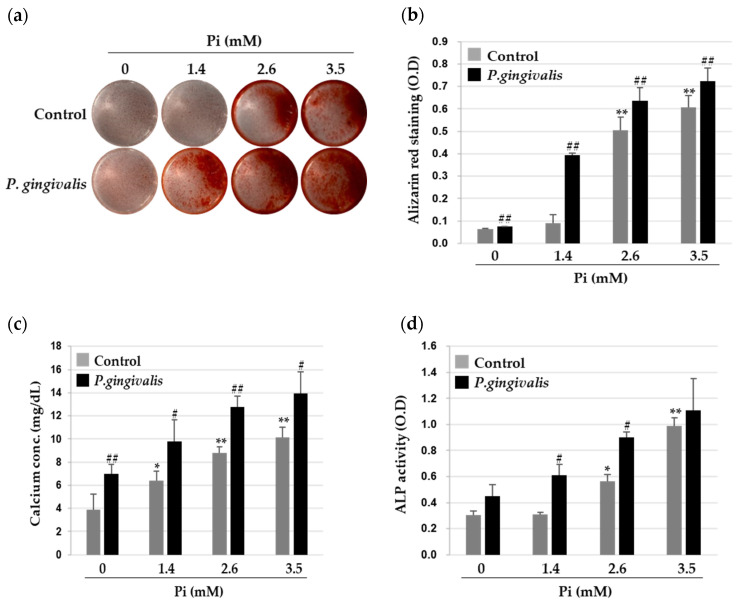
Effect of *P. gingivalis* in inorganic phosphate (Pi)-induced calcium deposition in vascular smooth muscle cells (VSMCs). A7r5 cells were infected with *P. gingivalis* for 3 h and cultured for five days in calcification medium containing 0, 1.4, 2.6, and 3.5 mM Pi. (**a**,**b**) Calcium deposition was stained with Alizarin Red S (left), and absorbance was measured to evaluate the degree of mineralization (right). (**c**) Calcium content was measured by a colorimetric calcium assay. (**d**) Alkaline phosphatase activity was measured and normalized to protein content for quantitative analysis. * *p* < 0.05; ** *p* < 0.01 vs. 0 mM Pi, # *p* < 0.05; ## *p* < 0.01 vs. control, one-way ANOVA followed by a Student’s t-test. Data shown are the mean ± SD, obtained for at least three independent experiments.

To study how *P. gingivalis* modulates VSMC calcification, we first identified the internalization of *P. gingivalis* into A7r5 cells, using CFSE-labeled *P. gingivalis*, and confirmed it by real-time RT-PCR. The internalization and expression of *P. gingivalis* factors in A7r5 cells were increased by *P. gingivalis* infection, whereas these were negligible in the non-infection group (Figure S1a,b). To investigate the effects of *P. gingivalis* on the Pi-induced VSMC calcification, *P. gingivalis*-infected A7r5 cells were treated with various concentrations of Pi for five days, and the extent of calcification was measured by Alizarin Red S (ARS) staining. As shown in Figure 1a,b, no calcium deposition occurred in A7r5 cells when incubated in 1.4 mM Pi, the human physiological concentration of serum phosphate, but dose-dependent calcium deposition was observed when the phosphate levels increased to 2.6 mM and 3.5 mM. Interestingly, *P. gingivalis*-infected A7r5 cells showed greater deposition of calcium than uninfected cells. Quantitative analysis indicated that the calcium content in the Pi-treated A7r5 cells increased significantly with *P. gingivalis* infection (Figure 1c). The activity of alkaline phosphatase (ALP), a molecular marker for vascular calcification, also showed a similar association with *P. gingivalis* infection. As shown in Figure 1d, while Pi induced significant ALP activity in A7r5 cells, this effect was markedly elevated upon *P. gingivalis* infection.

### 3.2. P.gingivalis Stimulates Pi-Induced Osteogenic Transdifferentiation of VSMCs

**Figure 2:**
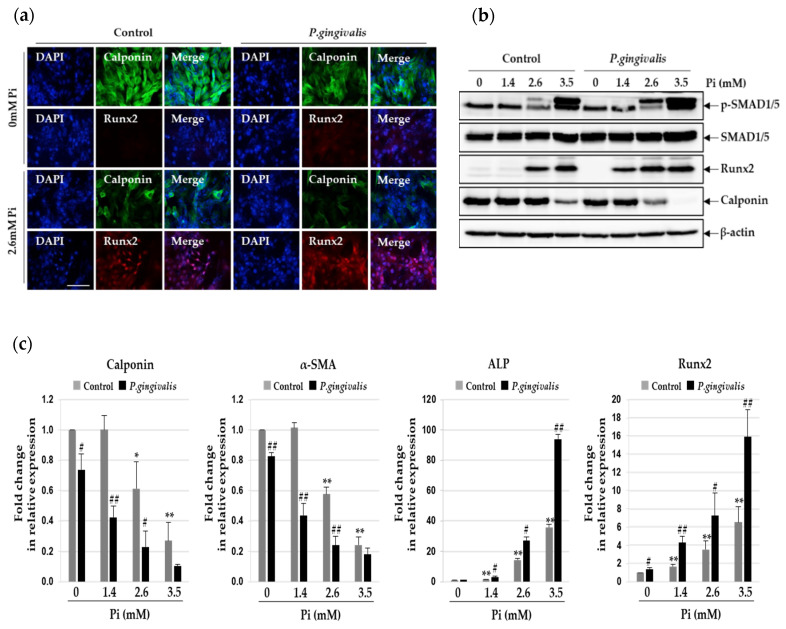
Effect of *P. gingivalis* on Pi-induced osteogenic transition in VSMCs. *P. gingivalis*-infected A7r5 cells were cultured for five days in calcification medium containing 2.6 mM Pi. (**a**) Higher magnification images showed immunoreactivity of Runx2 (red) and calponin (green) in *P. gingivalis*-infected cells under calcification conditions. (original magnification, ×400). Scale bar: 50 μm. (**b**) Protein levels of Runx2, calponin, p-SMAD1/5, and SMAD1/5 were examined by Western blotting using their corresponding antibodies. β-actin served as the loading control. (**c**) Total RNA was isolated and analyzed by real-time PCR using primers specific for rat Runx2, ALP, calponin, and α-SMA. The expression levels of the control (untreated) were set to 1, and the values were normalized to β-actin mRNA levels. * *p* < 0.05; ** *p* < 0.01 vs. 0 mM Pi, # *p* < 0.05; ## *p* < 0.01 vs. control, one-way ANOVA followed by a Student’s t-test. Data shown are the mean ± SD, obtained for at least three independent experiments.

Phenotypic alteration of VSMCs to display osteogenic characteristics is important for vascular calcification [16]. We evaluated the effects of *P. gingivalis* on this process and measured the expression of the markers of contractile and osteogenic phenotypes. As shown in Figure 2a,b, the expression of calponin, a contractile phenotype marker, in the A7r5 cells decreased upon exposure to 2.6 mM Pi and further upon *P. gingivalis* infection. In contrast, the expression of the osteogenic marker Runx2 increased in Pi-treated A7r5 cells and further in *P. gingivalis*-infected cells. Quantitative real-time PCR analysis revealed lower mRNA levels of calponin and α-SMA and higher mRNA levels of Runx2 and ALP in *P. gingivalis*-infected cells than in uninfected cells (Figure 2c). Phosphorylated Smad1/5, which forms a complex with Smad4, can move to the cell nucleus to activate downstream osteogenic genes, such as Runx2 [17]. Therefore, we tested whether *P. gingivalis* infection alters the phosphorylation status of SMAD1/5 protein in calcified VSMCs. As expected, Pi induced the phosphorylation of SMAD1/5 protein in VSMCs, and *P. gingivalis* infection increased this dramatically, compared to the levels seen in the control cells. 

### 3.3. P. gingivalis Increases Pi-Induced Apoptosis and Matrix Vesicle Release in VSMCs 

**Figure 3:**
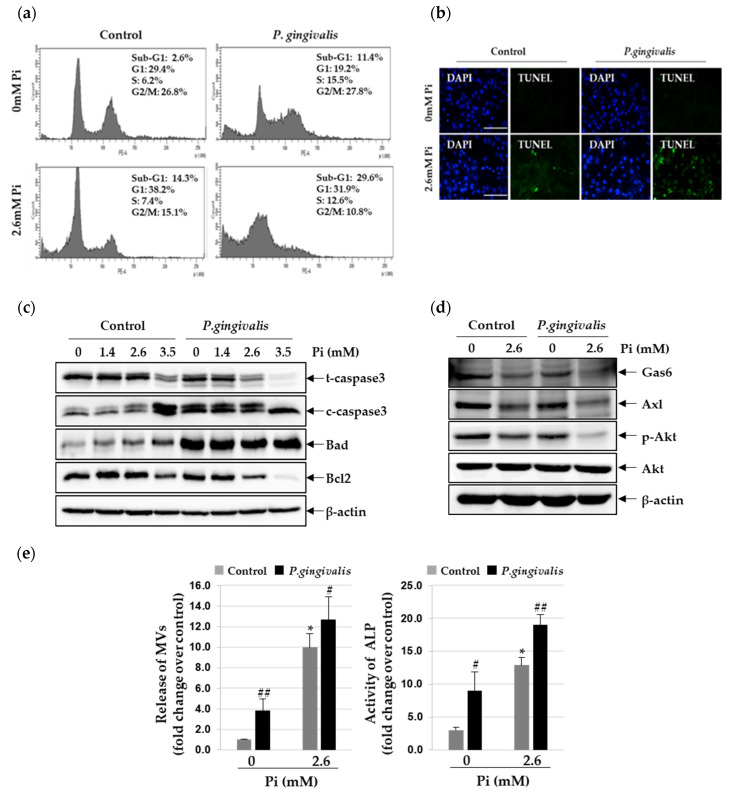
Effect of *P. gingivalis* on Pi-induced apoptosis and matrix vesicle release in VSMCs. *P. gingivalis*-infected A7r5 cells were cultured in calcification medium (2.6 mM Pi) for three days. (**a**) Induction of apoptosis was determined by flow cytometry analysis with PI staining. (**b**) Apoptosis-associated DNA fragmentation was detected by fluorescent TUNEL (green), and cell nuclei were stained by DAPI (blue). (original magnification, ×400). Scale bar: 50 μm. (**c**) Total/cleaved-caspase 3, Bcl2, Bad, and β-actin protein levels were examined by Western blotting using their corresponding antibodies. (**d**) Gas6, Axl, p-Akt, Akt, and β-actin protein levels were examined by Western blotting using their corresponding antibodies. (**e**) Matrix vesicles were isolated as described in the Materials and methods section. Alkaline phosphatase activity was measured and normalized to the total protein content of matrix vesicles. * *p* < 0.01 vs. 0 mM Pi, # *p* < 0.05; ## *p* < 0.01 vs. control, one-way ANOVA followed by a Student’s *t*-test. Data shown are the mean ± SD, obtained for at least three independent experiments.

Accumulating evidence suggests that apoptosis of VSMC promotes vascular calcification, primarily through the release of the calcifying membrane-bound matrix vesicles [18,19]. To understand whether these processes are involved in the *P. gingivalis*-induced vascular calcification, we assessed the status of apoptosis in *P. gingivalis*-infected VSMCs, using propidium iodide staining. *P. gingivalis* infection significantly increased the Pi-induced apoptosis of VSMCs (by 8.8~15.3% compared to that in non-infected cells) (Figure 3a). Apoptosis-induced DNA fragments were detected in the TUNEL assay. As shown in Figure 3b, TUNEL-positive apoptotic cells increased sharply when calcified VSMCs were infected with *P. gingivalis*. To explore the mechanism through which *P. gingivalis* enhances the Pi-induced apoptosis in VSMCs, the expression levels of various pro- and anti-apoptotic proteins were evaluated by western blot analysis. As shown in Figure 3c, the level of cleaved caspase-3 and Bad increased due to exposure to high concentrations of Pi and even further upon *P. gingivalis* infection. We also found that *P. gingivalis* infection drastically reduces the expression of Bcl2 protein in calcified VSMCs. Inorganic phosphate has been shown to induce apoptosis and calcification of VSMCs through inhibiting Gas6/Axl/Akt survival pathway [20]. Therefore, we examined whether the Gas6/Axl/Akt survival pathway was altered during Pi-induced apoptosis. We found that exposure to 2.6 mM Pi markedly down-regulated the expression of Gas6 and Axl and led to Akt inactivation, and this suppression was far more significant in *P. gingivalis*-infected cells (Figure 3d). Next, through collagenase digestion, matrix vesicles were isolated from the lysates of calcified VSMCs with and without *P. gingivalis* infection. As shown in Figure 3e, 2.6 mM Pi stimulated the release of matrix vesicles by more than 9.5 fold compared to control, and this was further stimulated by *P. gingivalis* infection. A similar trend was also seen in the levels of ALP activity. 

### 3.4. P. gingivalis Induces Vascular Calcification in Aortic Culture Ex Vivo

**Figure 4:**
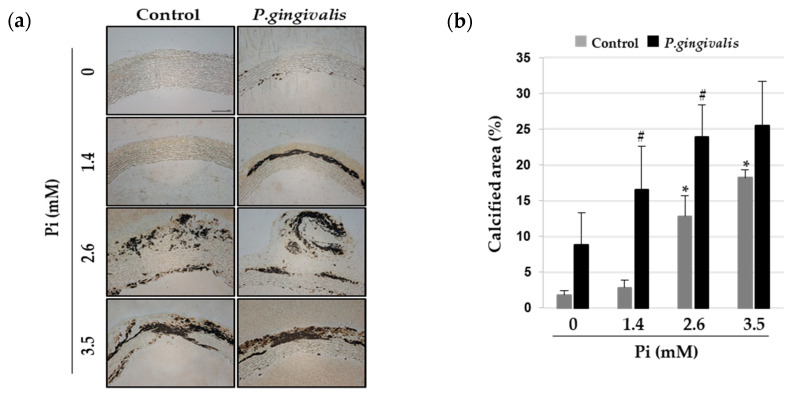

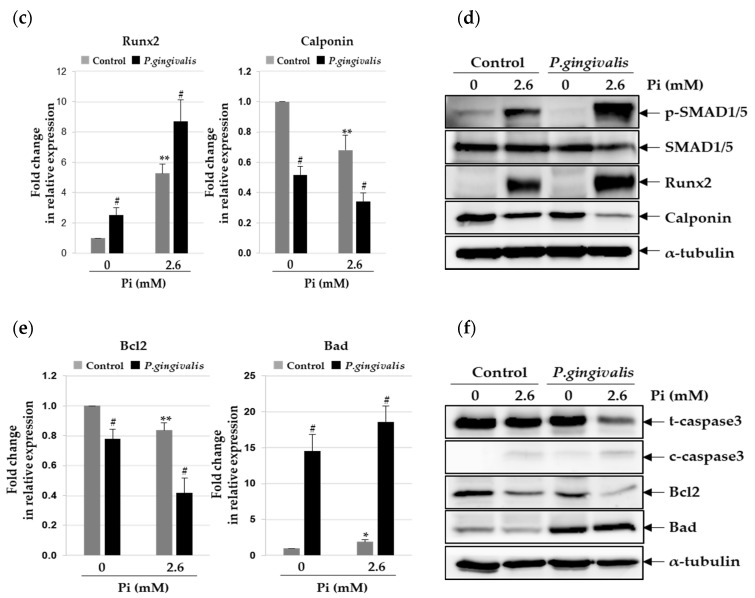
Effect of *P. gingivalis* on Pi-induced vascular calcification in aortic culture. *P. gingivalis*-infected rat aortas were cultured in calcification medium (2.6 mM) for eight days. (**a**) Calcified lesions were examined by von Kossa staining. Magnification is 200×. (**b**) The percentage of the calcified area was calculated using Calcification Analyzer Ver2. * *p* < 0.01 vs. 0 mM Pi, # *p* < 0.05 vs. control, one-way ANOVA followed by a Student’s t-test. (**c**,**e**) Total RNA was isolated from calcified rat aortas and analyzed by real-time RT-PCR using primers specific for rat Runx2, calponin, Bcl2, and Bad. The expression level of the control (untreated) was set to 1, and the values were normalized to the β-actin mRNA levels. * *p* < 0.05; ** *p* < 0.01 vs. 0 mM Pi, # *p* < 0.01 vs. control, one-way ANOVA followed by a Student’s t-test. Data shown are the mean ± SD, obtained for at least three independent experiments. (**d**,**f**) Phospho-SMAD1/5, SMAD1/5, Runx2, calponin, total/cleaved-caspase3, Bcl2, Bad, and α-tubulin protein levels were examined by Western blotting using their corresponding antibodies.

The organ culture of the aorta is useful for the ex vivo study of vascular calcification [21]. To test whether *P. gingivalis* promotes vascular calcification in rat aortic rings, sections of the thoracic aorta were infected with *P. gingivalis* and cultured in calcification medium with different concentrations of Pi for eight days. Morphometric assessment of calcium deposition was done by von Kossa staining. As shown in Figure 4a, calcium deposition was seen in VSMC layers of the aorta upon exposure to 2.6 mM and 3.5 mM Pi. Interestingly, *P. gingivalis*-infected aorta showed von Kossa-stained areas in the presence of 1.4 mM Pi, which is the normal physiological concentration of serum phosphate [22]. The ratio of calcified tissue area to normal tissue area was elevated in aortic explants due to *P. gingivalis* infection (Figure 4b). Next, we evaluated the effects of *P. gingivalis* infection on osteogenic differentiation and apoptosis of calcified aorta. Real-time RT-PCR and Western blot analyses revealed that *P. gingivalis* boosted the Pi-induced reduction in calponin level and increased Runx2 and phosphorylated SMAD1/5 levels (Figure 4c,d). Furthermore, the Pi-induced down-regulation of Bcl2 and up-regulation of cleaved-caspase3 and Bad were more pronounced with *P. gingivalis* infection (Figure 4e,f). 

## 4. Discussion

A typical pathological feature of patients with chronic kidney disease (CKD) is accelerated vascular calcification, which increases the risk and complicates the management of cardiovascular morbidities [23]. A strong association of CKD with periodontal infections is also reported [24]. Elevated levels of serum antibody to *P. gingivalis* are positively linked to loss of kidney function in a community-based cohort of elderly Japanese individuals [25]. However, the pathogenic mechanism by which the periodontal pathogens, particularly *P. gingivalis*, induce renal dysfunction and CKD, is not known. We are currently investigating whether serum levels of antibody to *P. gingivalis* correlate with the prevalence and extent of calcification of the arteries in patients with severe chronic renal impairment.

The pathological process of vascular calcification is actively regulated by diverse inducers and inhibitors, which are possibly driven by developmental, inflammatory, or metabolic factors [26]. In the present study, we observed that *P. gingivalis* promotes the transdifferentiation of VSMCs into osteoblast-like cells at higher concentrations of Pi, typically observed in hyperphosphatemic individuals. Several factors, including elevated phosphate levels, bone morphogenetic proteins, oxidative stress, and inflammatory cytokines, drive the vascular calcification [27]. Similarly, inflammatory cytokines, such as tumor necrosis factor (TNF)-α and interleukin (IL)-1β induce vascular calcification by up-regulating ALP and RUNX2 in VSMCs [14,28]. It is possible that the cytokines released by the *P. gingivalis*-stimulated VSMCs or *P. gingivalis* itself promote the Pi-induced vascular calcification. Down-regulation of the inhibitors of vascular mineralization, such as matrix Gla protein or fetuin-A, is associated with increased vascular calcification and consequent mortality due to cardiovascular dysfunction in patients with CKD [29,30]. Serum or salivary fetuin-A levels are known to decrease significantly with increasing severity of periodontal diseases [31,32]. Further investigations are needed to determine if and how this process involves fetuin-A, which serves as a marker of periodontal pathogen-stimulated vascular calcification or as a mediator linking periodontal diseases with vascular calcification. 

It is not known how periodontal bacteria gain access to the bloodstream, attach to the vascular wall, and invade vascular cells, including arterial endothelial cells and arterial SMCs [12,13]. Considering the epidemiological evidence to support an association between periodontal infections and atherosclerosis, there have been attempts to demonstrate the presence of periodontal pathogens, such as *P. gingivalis*, or their components in the atherosclerotic lesions [33]. Genomic DNA of some periodontal pathogens was isolated from the atheromatous samples from patients with periodontal diseases [34,35]. Viable *P. gingivalis* pathogens have also been detected in human atherosclerotic plaques with fluorescence in situ hybridization and invasion assays, indicating that active *P. gingivalis* can invade and survive in atherosclerotic plaques [36,37]. VSMCs are one of the major components of the vessel walls and are involved in the development and progression of atherosclerosis [38]. There are only a few studies to show the pathogenic association between VSMCs that are challenged with live *P. gingivalis* and the possible signaling mechanisms involved in atherosclerosis. *P. gingivalis* is reported to invade human aortic SMCs and stimulate their proliferation through activation of TGF-β and Notch signaling [39]. Under hyperglycemia, *P. gingivalis* infection initiates vascular calcification in human aortic SMCs by autocrine regulation of BMP4 [40].

Gingival crevicular fluid (GCF), found in the gingival sulcus surrounding the teeth, is in close proximity to the periodontal tissues, leading to provide more substantial information about periodontal disease than saliva [41]. Since different levels of inflammatory mediators and periodontogens have been simply detected and monitored in GCF, it is an ideal strategy to reflect the severity of periodontitis and to detect the interaction between periodontogens and host [42,43]. The molecular analysis of GCF components may assist in defining how periodontal disease has an effect on development and progression of systemic diseases including vascular calcification and cardiovascular disease. 

In the present study, we showed the effect of *P. gingivalis* infection on the Pi-induced vascular calcification induced by matrix vesicle release, apoptosis, and osteogenic differentiation of VSMCs in vitro and vascular calcification of arterial rings ex vivo, and elucidated the mechanisms involved in their calcification. Our findings provide opportunities to understand the potential role of periodontal pathogen infection as a risk factor for vascular diseases associated with vascular calcification.

## Supplementary Materials

The following are available online at https://www.mdpi.com/2073-4409/9/12/2694/s1, Figure S1: Effect of *P. gingivalis* infection in A7r5 cells, Table S1: Primer sequences for real-time RT-PCR.
