# Supplementary material for: Infection of Porphyromonas gingivalis Increases Phosphate-Induced Calcification of Vascular Smooth Muscle Cells
Source: Cells. 2020 Dec 15;9(12):2694. doi: 10.3390/cells9122694 (PMC7765351; doi:10.3390/cells9122694)
Supplement: Supplementary file 1 [file cells-09-02694-s001.pdf]

Infection of *Porphyromonas gingivalis* increases phosphate-induced calcification of vascular smooth muscle cells

Hyun-Joo Park[1,4,5], Yeon Kim[1,4,5], Mi-Kyoung Kim[1,4], Hae Ryoun Park[2,4], Hyung Joon Kim[1,4], Soo-Kyung Bae[3,4], and Moon-Kyoung Bae[1,4,5*]

[1] Department of Oral Physiology, BK21 PLUS Project, School of Dentistry, Pusan National University, Yangsan 50610, South Korea; phj3421@hanmail.net (H.-J.P.); graceyeon88@gmail.com (Y.K.) eenga@naver.com (M.-K.K.); hjoonkim@pusan.ac.kr (H.J.K)
[2] Department of Oral Pathology, School of Dentistry, Pusan National University, Yangsan 50610, South Korea; parkhr@pusan.ac.kr (H.R.P.)
[3] Department of Dental Pharmacology, BK21 PLUS Project, School of Dentistry, Pusan National University, Yangsan 50610, South Korea; skbae@pusan.ac.kr (S.-K.B)
[4] Periodontal Disease Signaling Network Research Center (MRC), School of Dentistry, Pusan National University, Dental Research Institute, Yangsan 50610, South Korea
[5] Dental and Life Science Institute, School of Dentistry, Pusan National University, Yangsan 50610, South Korea
* Correspondence: mkbae@pusan.ac.kr; Tel.: +82-51-510-8239

Supplementary Information

The following file contains supplementary material for the paper "Infection of *Porphyromonas gingivalis* increases phosphate-induced calcification of vascular smooth muscle cells".

This file is composed of:

- Supplementary figures and relative supplementary figure legends
- Supplementary tables

Supplementary Figures

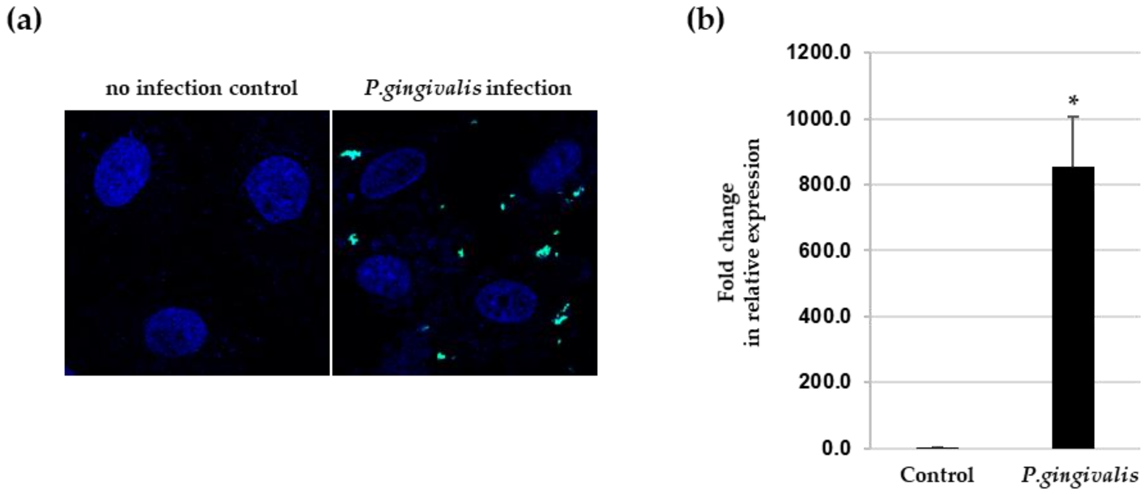

Figure S1. Effect of *P.gingivalis* infection in A7r5 cells. (A) A7r5 cells were infected with carboxyfluorescein succinimidyl ester (CFSE)-stained *P. gingivalis* for 3 h and then observed under a fluorescent microscope. (B) Using real-time RT-PCR, the presence of *P.gingivalis* was also confirmed. *P<0.001 vs. control. Data shown are the mean ± SD, obtained for at least three independent experiments.

Supplementary Tables

Table S1. Primer sequences for real-time RT-PCR

Genes	Sequences (5′ → 3′)	Length(bp)
P.gingivalis	Forward: TCGGTAAGTCAGCGGTGAAAC<br>Reverse: GCAAGCTGCCTTCGCAAT	150bp
β-actin	Forward: AGGGAAATCGTGCGTGAC<br>Reverse: CGCTCATTGCCGATAGTG	146bp
Runx2	Forward: GCCGGGAATGATGAGAACTA<br>Reverse: TGGGGAGGATTTGTGAAGAC	155bp
ALP	Forward: TGCTTTGTGTGTGCTGACTGTA<br>Reverse: AGTGACGGTGTCGTAGCCTTC T	129bp
calponin	Forward: GAACAAGCTGGCCCAGAAAT<br>Reverse: GGCCATCCATGAAGTTGCTC	104bp
α-SMA	Forward: AAGAGTTACGAGTTGCCTGATG<br>Reverse: TGATGCTGTTGTAGGTGGTTT	136bp
Bcl2	Forward: GCCCTGTGGATGACTGAGTA<br>Reverse: CAGGTATGCACCCAGAGTGA	189bp
Bad	Forward: AGGGATGGAGGAGGAGCTTA<br>Reverse: GGAACCCTCAAACTCATCGC	121bp
